# Genome-Wide Association Analyses in the Model Rhizobium *Ensifer meliloti*

**DOI:** 10.1128/mSphere.00386-18

**Published:** 2018-10-24

**Authors:** Brendan Epstein, Reda A. I. Abou-Shanab, Abdelaal Shamseldin, Margaret R. Taylor, Joseph Guhlin, Liana T. Burghardt, Matthew Nelson, Michael J. Sadowsky, Peter Tiffin

**Affiliations:** a Department of Plant and Microbial Biology, University of Minnesota, Saint Paul, Minnesota, USA; b Biotechnology Institute, University of Minnesota, Saint Paul, Minnesota, USA; c Environmental Biotechnology Department, Genetic Engineering and Biotechnology Research Institute (GEBRI) at City of Scientific Research and Technology Applications (SRTA-City) New Borg El Arab, Alexandria, Egypt; d Department of Soil, Water, and Climate, University of Minnesota, Saint Paul, Minnesota, USA; The Jackson Laboratory for Genomic Medicine

**Keywords:** BSLMM, GWAS, *Medicago*, rhizobium, *Sinorhizobium*, bacteria, chip heritability, genetic architecture, genomics, linkage disequilibrium, symbiosis

## Abstract

Genome-wide association studies (GWAS) can identify genetic variants responsible for naturally occurring and quantitative phenotypic variation. Association studies therefore provide a powerful complement to approaches that rely on *de novo* mutations for characterizing gene function. Although bacteria should be amenable to GWAS, few GWAS have been conducted on bacteria, and the extent to which nonindependence among genomic variants (e.g., linkage disequilibrium [LD]) and the genetic architecture of phenotypic traits will affect GWAS performance is unclear. We apply association analyses to identify candidate genes underlying variation in 20 biochemical, growth, and symbiotic phenotypes among 153 strains of Ensifer meliloti. For 11 traits, we find genotype-phenotype associations that are stronger than expected by chance, with the candidates in relatively small linkage groups, indicating that LD does not preclude resolving association candidates to relatively small genomic regions. The significant candidates show an enrichment for nucleotide polymorphisms (SNPs) over gene presence-absence variation (PAV), and for five traits, candidates are enriched in large linkage groups, a possible signature of epistasis. Many of the variants most strongly associated with symbiosis phenotypes were in genes previously identified as being involved in nitrogen fixation or nodulation. For other traits, apparently strong associations were not stronger than the range of associations detected in permuted data. In sum, our data show that GWAS in bacteria may be a powerful tool for characterizing genetic architecture and identifying genes responsible for phenotypic variation. However, careful evaluation of candidates is necessary to avoid false signals of association.

**IMPORTANCE** Genome-wide association analyses are a powerful approach for identifying gene function. These analyses are becoming commonplace in studies of humans, domesticated animals, and crop plants but have rarely been conducted in bacteria. We applied association analyses to 20 traits measured in Ensifer meliloti, an agriculturally and ecologically important bacterium because it fixes nitrogen when in symbiosis with leguminous plants. We identified candidate alleles and gene presence-absence variants underlying variation in symbiosis traits, antibiotic resistance, and use of various carbon sources; some of these candidates are in genes previously known to affect these traits whereas others were in genes that have not been well characterized. Our results point to the potential power of association analyses in bacteria, but also to the need to carefully evaluate the potential for false associations.

## Abstract (precis)

Genome-wide association analyses are a powerful approach for identifying gene function. These analyses are becoming commonplace in studies of humans, domesticated animals, and crop plants but have rarely been conducted in bacteria. We applied association analyses to 20 traits measured in Ensifer meliloti, an agriculturally and ecologically important bacterium because it fixes nitrogen when in symbiosis with leguminous plants. We identified candidate alleles and gene presence-absence variants underlying variation in symbiosis traits, antibiotic resistance, and use of various carbon sources; some of these candidates are in genes previously known to affect these traits whereas others were in genes that have not been well characterized. Our results point to the potential power of association analyses in bacteria, but also to the need to carefully evaluate the potential for false associations.

## INTRODUCTION

Identifying gene function in bacteria has largely relied on forward or reverse genetics. In their standard application, these approaches rely on *de novo* mutations, and often mutations that cause complete loss of function. Association analyses, or genome-wide association studies (GWAS), provide a complementary approach for identifying gene function by using statistical approaches to associate naturally occurring allelic variation with phenotypic variation (1). Unlike approaches that rely on *de novo* mutations, GWAS identify segregating alleles that are responsible for naturally occurring phenotypic variation, the variation that has resulted from and is the raw material for evolution. Identifying naturally segregating phenotype-associated alleles can inform fundamental questions about genotype-to-phenotype mapping, such as the role of regulatory versus coding variants (e.g., reference 2), rare versus common variants (e.g., references 3 and 4), SNPs versus structural variants (e.g., reference 5), and the effect sizes of causative variants (e.g., references 6 and 7).

GWAS have been used to explore the genetic basis of phenotypic variation in humans (7), model eukaryotes (e.g., references 8 and 9), and domesticated plants and animals (10, 11). Because bacterial genomes can be sequenced relatively inexpensively and phenotypes can be readily measured under controlled conditions, bacteria could be highly amenable to association analyses (12–14). In fact, there are several examples of GWA methods successfully being applied to bacteria. GWAS have identified both previously known and novel operons associated with nickel tolerance in *Mesorhizobium* (15), copy-number variants associated with alginate metabolism in *Vibrio* (16), presence-absence variation (PAV) of known and novel virulence factors associated with infectivity in *Listeria* (17), antibiotic resistance genes in several lineages (18, 19), and both gene presence-absence and nucleotide variants associated with host range of *Campylobacter* (20).

Despite the potential for bacterial GWAS, the nonindependence of segregating variants, i.e., linkage disequilibrium (LD), may be problematic. Recombination in prokaryotes typically operates by gene conversion or double recombination (reviewed in reference 21). Thus, LD will not necessarily decay monotonically with genomic distance as it is generally assumed to do in eukaryotic species (22). Recombination rates vary widely both among bacterial lineages and within species (23, 24), in part due to variation among species in transformation competence and DNA repair machinery (25) or population structure (26). The extent of recombination and relatedness might limit the ability of GWAS to pinpoint the specific variants responsible for phenotypic variation and thus is important to consider when choosing statistical approaches to conduct association analyses in bacteria (14, 18, 27).

Ensifer meliloti (formerly Sinorhizobium meliloti) is an ecologically and agriculturally important species that has been extensively studied. Much of the work on *Ensifer* has been motivated by its role as a facultative symbiont of legumes, primarily *Medicago* species. As a symbiont, *Ensifer* converts atmospheric N_2_ into a plant-usable form, thereby providing plants with an essential nutrient and contributing to plant growth and productivity (28). Because of the importance of N-fixation, most genetic analyses of *Ensifer* have focused on genes responsible for the establishment and function of symbiosis (reviewed in reference 29). These analyses have identified genes responsible for attraction of rhizobia to plant roots, nodule establishment, and N-fixation (30). However, which of these or other genes are segregating allelic variation responsible for phenotypic variation in nature is an open question. Moreover, the genetic basis of other traits that might be important in *Ensifer* ecology and survival outside the host has not been well studied.

The primary objectives of this work were to evaluate the performance of phenotype-genotype mapping in bacteria and to advance our understanding of the genetic basis of phenotypic variation in *Ensifer*. We pursued these objectives using a collection of 153 strains of *E. meliloti*. We fully sequenced each strain to identify single nucleotide polymorphisms (SNPs) and gene presence absence variants (PAVs) and phenotyped for 20 diverse symbiotic, metabolic, growth, and environmental tolerance traits. Because the performance of association mapping depends on the population genomic characteristics of the sample, we first characterized genomic diversity and genome-wide LD. We then used association analyses to characterize the number, minor allele frequency, and effect sizes of variants contributing to phenotypic variation, using permutations to identify those variants that had greater contribution than expected by chance.

## RESULTS

We used the Illumina platform to sequence 153 Ensifer meliloti strains to a mean depth of 19.5×, and then aligned reads to *E. meliloti* strain USDA1106 to identify genomic variants. Across the three main replicons of the *E. meliloti* genome (∼3.5-Mb chromosome, ∼1.8-Mb megaplasmid pSymB, and ∼1.5-Mb megaplasmid pSymA), we identified 439,288 segregating sites with <20% missing data, 96.2% of which were biallelic, and 66,283 annotated genes that varied in whether they were present or absent (PAVs) (706 genes were present in all strains, 1,219 genes were present in all but one strain, and 31,787 genes were present in only one strain). Of these variants, 123,955 (110,603 SNPs and 13,352 PAVs) had a minor allele frequency (MAF) >5%. Consistent with previous characterizations of genomic diversity in *Ensifer* (e.g., references 31 and 32), nucleotide diversity was less on the chromosome (196,881 SNPs, 1 per 19 bp, θ_W_ = 0.010, θ_π_ = 0.004) than either pSymB (146,268 SNPs, 1 per 11 bp, θ_W_ = 0.016, θ_π_ = 0.009) or pSymA (96,139 SNPs, 1 per 14 bp, θ_W_ = 0.013, θ_π_ = 0.009). Similar patterns were obtained when including only those variants with MAF > 0.05 (chromosome θ_W_ = 0.002, θ_π_ = 0.003; pSymB θ_W_ = 0.005, θ_π_ = 0.008; pSymA θ_W_ = 0.006, θ_π_ = 0.008). Most (10,318) of the common (MAF > 0.05) PAVs were not present in the reference genome, and so we did not attempt to determine their genomic location. Of the PAVs that were present in the reference, the replicon with the greatest proportion of variable genes was megaplasmid pSymA (54% of the genes on pSymA, 860 genes), followed by pSymB (46%, 780 genes), and the chromosome (37%, 1,394 genes).

**FIG 1:**
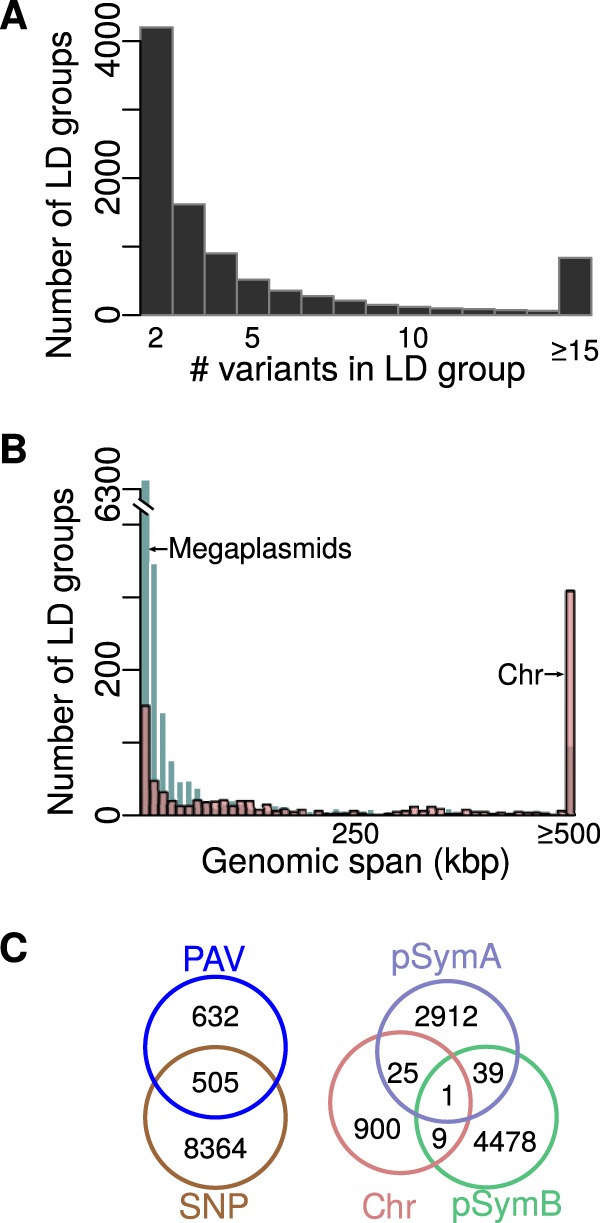
(A) Distribution of number of variants per LD group (at *r*^2^ ≥ 0.95), (B) distribution of genomic distance spanned by LD groups found on the chromosome or the megaplasmids (including only groups found only on one replicon), and (C) number of groups containing only PAVs, only SNPs, or both as well as the number of LD groups found within and across replicons. There were 22,057 SNPs and 10,674 PAVs that were not grouped with other variants and 9,501 LD groups with a median of three variants per group, and the largest group contained 6,970 variants. Half of all variants are in groups that contain ≤12 variants. Only variants used for association testing (minor allele frequency ≥ 5%, missingness ≤ 20%) were grouped.

Variants in strong linkage disequilibrium (LD) are nonindependent and thus statistically indistinguishable in association analyses (e.g., reference 33). To identify nonindependence among the ∼124,000 common variants (MAF > 0.05), we identified groups of variants in high LD with one another. At an LD threshold of *r*^2^ ≥ 0.95, approximately 20% of the SNPs (22,057) and 80% of the PAVs (10,764) were not grouped with any other variants. However, the majority of variants were strongly linked to one or more other variants. The median size of these groups was three variants, and most groups contained only SNPs (8,364 groups) or only PAVs (632 groups) (Fig. 1), indicating that association analyses conducted only on SNPs are unlikely to identify PAVs responsible for phenotypic variation and vice versa.

LD tended to be more extensive on the chromosome than either megaplasmid. The mean *r*^2^ between pairs of SNPs was 0.24 on the chromosome, 0.05 on pSymB, and 0.12 on pSymA (Table 1). Chromosomal LD groups with more than one SNP contained a median of seven variants and spanned a median distance of ∼170 kbp (Table 1). In contrast, on pSymB and pSymA, the median group size of groups with more than one variant was three variants and the median spanned distance was 500 to 1,000 bp (Table 1 and Fig. 1; see also Fig. S1C in the supplemental material). Thus, while recombination has shaped the distribution of segregating variation on the chromosome, it has played a larger role on the megaplasmids. At a relaxed LD threshold of *r*^2^ ≥ 0.80, there were fewer LD groups, but the distributions of LD group size and types of variants contained were similar (Fig. S1A and B).

**TABLE 1:** Mean *r*^2^, a measure of nonindependence between segregating variants, is generally low between pairs of variants of different types or on different replicons, while the median size and spanned distance of LD groups is less on the megaplasmids than on the chromosome

Variant type or location	Mean *r*^2^ between variants	No. ungrouped variants	No. of LD groups	Median no. of variants per LD group	Median LD group spanned distance^*a*^
All	0.06	32,821	9,501	3	N/A
SNPs only	0.07	22,057	8,364	3	N/A
PAVs only	0.02	10,764	632	2	N/A
Between SNPs and PAVs	0.03	N/A	505	7	N/A
Chromosome SNPs	0.24	789	900	7	173,406
pSymB SNPs	0.05	13,671	4,478	3	518
pSymA SNPs	0.12	7,597	2,912	3	1,063

a Spanned distance calculated only for LD groups with SNPs that were all on the same replicon.

10.1128/mSphere.00386-18.2 FIG S1 (A) Distribution of number of variants in each LD group at *r*^2^ = 0.80. There were 27,509 LD groups total, and half of the variants were found in LD groups with 37 or fewer variants. (B) Number of LD groups containing PAVs or SNPs, and number of LD groups containing SNPs found on each replicon, also at *r*^2^ = 0.80. (C) Distributions of number of variants per LD group for LD groups at *r*^2^ = 0.95 containing only SNPs, only PAVs, or only SNPs on one of the replicons. Download FIG S1, PDF file, 1.3 MB. Copyright © 2018 Epstein et al. 2018 Epstein et al. This content is distributed under the terms of the Creative Commons Attribution 4.0 International license.

### Phenotypic variation explained.

**FIG 2:**
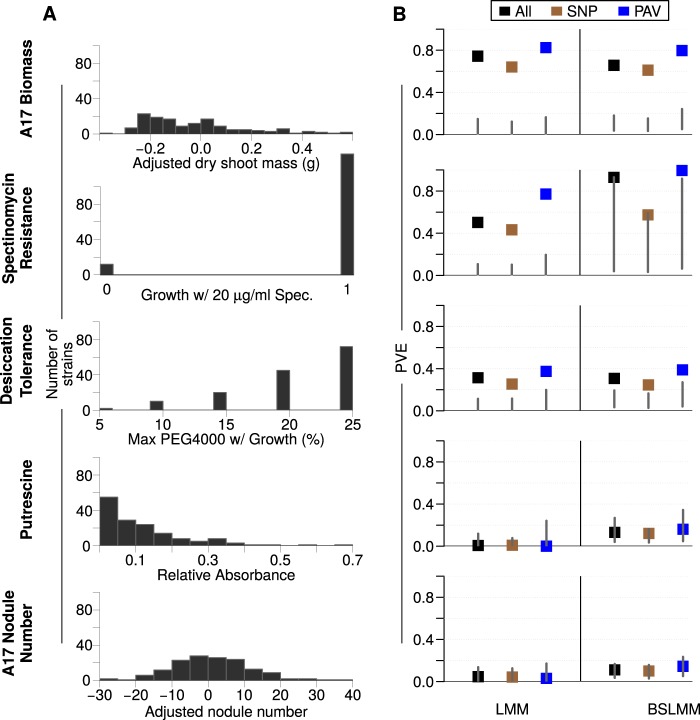
(A) Phenotypic distributions of the focal traits and (B) proportion of phenotypic variance explained (PVE) by relatedness among strains (i.e., the K-matrix) alone, as predicted by a linear mixed model, and by both relatedness and large-effect variants through a Bayesian sparse linear mixed model (BSLMM) implemented in GEMMA. PVE was calculated for all variants, only SNPs, and only PAVs. The gray lines indicate the lower 95% of the empirical null distributions from permuted data sets.

Association analyses are more likely to identify genes responsible for variation of Mendelian traits than for traits with continuous variation determined by genetic variation at many loci with small effects (6). Given this expectation, we selected five focal traits that capture a range of trait types and the range of phenotypic distributions among the studied 20 traits (Fig. S2): the distribution of plant biomass was approximately uniform, nodule number was approximately normal, putrescine metabolism was truncated normal, spectinomycin resistance was binary, and desiccation tolerance was multimodal (Fig. 2A). To estimate the proportion of phenotypic variation explained (PVE) by genetic differences rather than microenvironmental differences the strains experienced during the phenotypic assays, we applied two “chip heritability” (34) approaches to each trait. First, we used a linear mixed model (LMM) that assumes effect sizes are normally distributed and all variants contribute to variation. In essence, this method estimates how much phenotypic variation can be explained by genetic relatedness among strains and should be powerful at explaining variation in highly polygenic traits. Second, we used a Bayesian sparse linear mixed model (BSLMM) which includes a small effect for each variant, much like the LMM, as well as larger effects for a limited number of variants (35). The BSLMM should be more powerful than the LMM, because it can explain variation in highly polygenic traits as well as traits with a few genes of large effect. Estimates of PVE differed widely among these traits, ranging from nearly 100% for spectinomycin resistance to <10% for putrescine utilization. For all traits except spectinomycin resistance, the LMM and BSLMM estimates were similar (Fig. 2B), suggesting either that the traits are controlled by many small effect variants or that there are large effect variants but they are found in closely related strains.

10.1128/mSphere.00386-18.3 FIG S2 Distributions for all 20 phenotypic traits. The five focal traits are in red, and the 11 traits with PVE > 0 are in bold. Download FIG S2, PDF file, 0.3 MB. Copyright © 2018 Epstein et al. 2018 Epstein et al. This content is distributed under the terms of the Creative Commons Attribution 4.0 International license.

To verify that the estimates of PVE exceed what is expected by chance, we created 100 permuted data sets in which we randomly assigned phenotypic values to genotypes and reran the PVE analyses, resulting in an empirical null distribution of PVE estimates. For nearly all traits, genetic variation explained <25% of the variation in the permuted phenotypes (Fig. 2B). However, for spectinomycin resistance the upper bound of the BSLMM null distribution, but not the LMM null distribution, was nearly 100%, suggesting that BSLMM is overestimating PVE. This overestimate might be because there are relatively few spectinomycin-sensitive strains, resulting in a high probability that there are noncausative variants having alleles that match the distribution of resistance-sensitivity. The overestimate also indicates the need for cautious interpretation of PVE estimates for highly imbalanced phenotypic data.

### Single-variant associations.

The PVE analyses estimate the proportion of phenotypic variation that has a genetic basis but do not identify the causal variants. To identify causal variants, we conducted single-variant association analyses in which a measure of pairwise strain relatedness, the K-matrix, is used as a covariate and the effect of relatedness is statistically removed before testing each variant. Before analyzing the empirical data, we generated null expectations, i.e., empirical false discovery expectations, for the strength of variant-phenotype associations for each trait using the same data permutations we used to evaluate the performance of the LMM and BSLMM. Permutation tests are computationally intensive but produce null distributions that match the properties (e.g., trait distribution, amount of missing data, LD patterns) of each data set (36). The permuted data revealed that a substantial portion of phenotypic variation can be associated with genomic variants even when there is no true relationship between genotype and phenotype. The reason for this is simply that with more than 40,000 LD groups, one expects to find a considerable number of chance associations.

**FIG 3:**
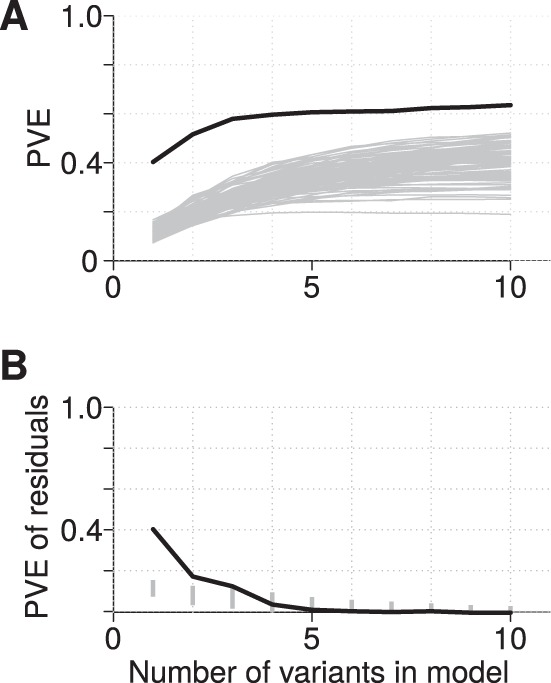
Evaluation of the expected proportion of variance explained (PVE) for A17 biomass by the most strongly associated variants as determined by association testing and forward model selection. Panel A shows the cumulative PVE explained by the 10 most strongly associated variants (black line, more than 10 variants rarely explained more variation than expected by chance) as well as the cumulative PVE from each of 100 randomly permuted data sets that make up the empirical null distribution (gray lines). For A17 biomass, the actual data explain more variance than the permuted data; however, panel B shows that only the first 3 variants explain more of the residual PVE (i.e., after accounting for PVE of the previous variants) than expected by chance. In panel B, the vertical gray lines represent the lower 95% of the null distribution.

**FIG 4:**
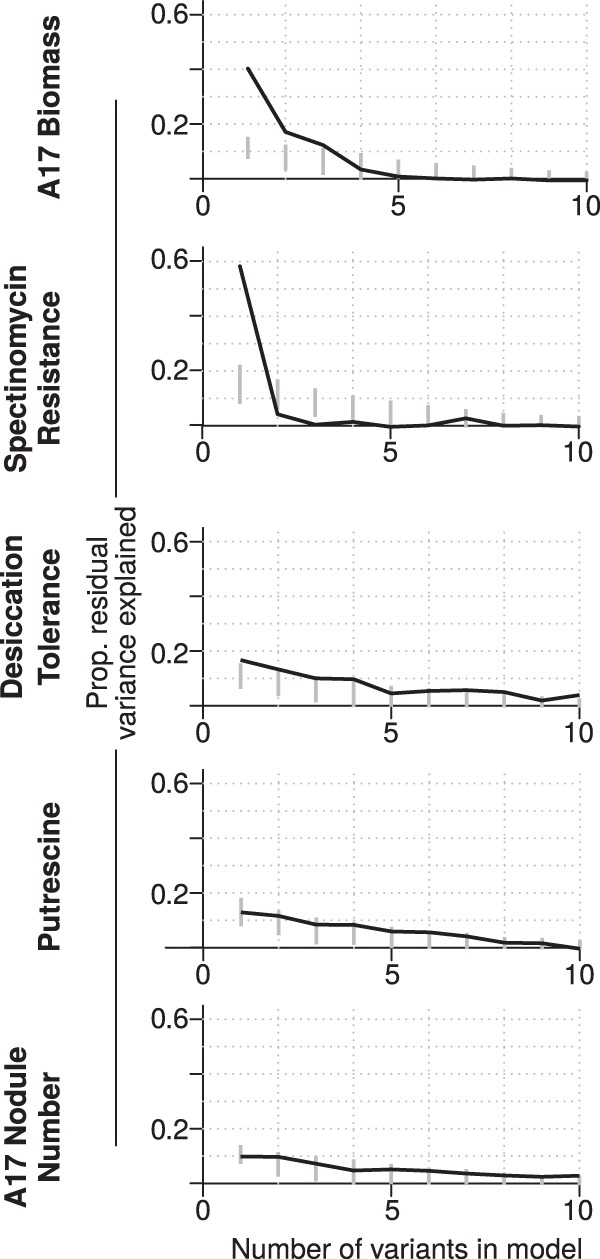
The proportion of remaining phenotypic variance of the focal traits explained by adding each additional top variant, as in Fig. 3B.

By comparing variants identified for each trait to the empirical null expectations, we determined which associations were stronger than expected by chance. For example, for A17 plant biomass (reflective of the symbiotic benefit each strain provides the host), the cumulative effects of the 10 most strongly associated variants explain more variation than is expected by chance (Fig. 3A); however, this cumulative effect is due primarily to only the top three variants (Fig. 3B). Similarly, among the other focal traits, only one variant clearly exceeds the null expectation for spectinomycin resistance and desiccation tolerance (Fig. 4). In contrast, for A17 nodule number and use of putrescine as a carbon source, the most strongly associated variants explain no more of the phenotypic variance than expected by chance (Fig. 4). We extended this analysis to the 15 nonfocal traits (Fig. S3) and found that seven had stronger genotype-phenotype associations than expected by chance (formic acid and 2-aminoethanol as C sources; gentamicin, streptomycin, biomass of the R108 host; and annual precipitation [AP] and annual mean temperature [AMT]). For all but two of these traits (2-aminoethanol as C source and AMT), the PVE by the polygenic modeling was also significantly greater than the empirical null expectation (Fig. S4), indicating that for half of the traits there was statistical power to identify candidate genes (Table 2). The number of nodules formed on the R108 host was unusual in that the most strongly associated variants did not explain more variation than the empirical null, but the polygenic modeling did. For all traits except use of 2-aminoethanol as C source, however, the polygenic model estimate of the PVE was greater than that from the top variants (Table 3), indicating that phenotypic variation also was influenced by undetected variants of small effect or unmodeled sources of genetic variation (e.g., copy number variants and epistatic interactions).

**TABLE 2:** Candidate genes tagged by variants in LD groups that explained more variation than expected based on the empirical null distribution (see Fig. S7 for QQ-plots)

Trait	Replicon	Position^*d*^	Annotation (MaGe^*a*^ locus tag)
2-Aminoethanol	pSymA	52580	*fixI*: nitrogen fixation protein FixI (SMEL_v1_mpb0065)
Formic acid	pSymA	38256	*napA*: nitrate reductase, periplasmic, large subunit (SMEL_v1_mpb0048)
Gentamicin	pSymA	796714	Transcriptional regulator, ROK family (SMEL_v1_mpb0963)
	pSymA	263510	*nifH*: nitrogenase Fe protein (SMEL_v1_mpb0322)
	pSymA	282760	Putative aldehyde dehydrogenase (SMEL_v1_mpb0345)
Spectinomycin	pSymA	PAV	Conserved protein of unknown function (SMEL_v1_mpb0259)
Streptomycin	Chrom.	PAV	Multisensor signal transduction histidine kinase (SMEL_v1_0575)
Desiccation	pSymB	1161576	Putative aldehyde or xanthine dehydrogenase (SMEL_v1_mpa1160)
A17 biomass	pSymA	269841	*nifA*: Nif-specific regulatory protein (SMEL_v1_mpb0330)
		269869	
		270090	
		270096	
		270157	
		270283	
		270292	
	pSymA	271348	*nifA*: (SMEL_v1_mpb0330); unknown (SMEL_v1_mpb0331)
	pSymA	274195	Unannotated
	pSymA	276359	*gabD*: succinate-semialdehyde dehydrogenase I, NADP-dependent (SMEL_v1_mpb0338)
		276443	
		276563	
	pSymB	1231268	*queC*: 7-cyano-7-deazaguanine synthase (SMEL_v1_mpa1230)
	pSymB	1376015	Diguanylate cyclase/phosphodiesterase (SMEL_v1_mpa1374)
	pSymB	669804	Sulfotransferase family (SMEL_v1_mpa0678)
R108 biomass	pSymA	305290	*fixN*: cytochrome *c* oxidase subunit 1 homolog (SMEL_v1_mpb0374)
		305308	
		305353	
AMT^*b*^	pSymA	648346	Diguanylate cyclase/phosphodiesterase (SMEL_v1_mpb0802)
		649133	
AP^*c*^	pSymA	PAV	*fixS*: FixS2 nitrogen fixation protein (SMEL_v1_mpb0492)

a http://www.genoscope.cns.fr/agc/microscope/home/index.php.

b Annual mean temperature.

c Annual precipitation.

d Variants are sorted by genomic position, not ranking or LD group.

**TABLE 3:** For most traits, phenotypic variance explained by genome-wide relatedness (“PVE LMM”) was greater than the phenotypic variance explained by just the top variants

Trait	PVE top variants^*a*^	PVE LMM^*b*^
2-Aminoethanol	0.05	0.00
Gentamicin resistance	0.14	0.49
Spectinomycin resistance	0.43	0.50
Streptomycin resistance	0.34	0.58
Annual mean temperature	0.09	0.12
Annual precipitation	0.10	0.23
Formic acid	0.08	0.30
Desiccation tolerance	0.19	0.31
A17 biomass	0.33	0.74
R108 biomass	0.19	0.53
R108 nodule number	0.06	0.19

a Maximum cumulative PVE among 1 to 25 variants chosen by model selection after subtracting the median of the empirical null distribution obtained from random permutations.

b After subtracting median of the null distribution.

10.1128/mSphere.00386-18.4 FIG S3 PVE explained by top variants from GWA after model selection. As in Fig. 4, the points represent the proportion of remaining variance explained (“PRVE”) by adding each additional variant, and, for clarity, only the top 10 of 25 variants are shown. The gray lines indicate the lower 95% of PRVE estimates from analyses of phenotypes randomly permuted 100 times. The five focal traits have red titles, and the ten traits for which at least the first variant explained more phenotypic variance than expected by chance have bolded titles. Download FIG S3, PDF file, 0.9 MB. Copyright © 2018 Epstein et al. 2018 Epstein et al. This content is distributed under the terms of the Creative Commons Attribution 4.0 International license.

10.1128/mSphere.00386-18.5 FIG S4 PVE by LMM (the K-matrix alone) and by the BSLMM. The gray lines indicate the lower 95% of random permutations. Note that random permutations were only conducted for BSLMM on the five focal traits because of computational limitations. The five focal traits are in red, and the nine traits with PVE by either the K-matrix or BSLMM >95% of the random permutations are in bold. Download FIG S4, PDF file, 0.6 MB. Copyright © 2018 Epstein et al. 2018 Epstein et al. This content is distributed under the terms of the Creative Commons Attribution 4.0 International license.

For the ten traits for which we had power to identify candidate genes, we performed additional analyses. First, we compared the mean minor allele frequency (MAF) of the candidates to the MAF of the variants most strongly associated with the permuted data. This analysis revealed that candidates most strongly associated with variation in A17 biomass, and, to a lesser extent, annual mean temperature, were highly enriched for intermediate frequency variants (Fig. S5). We conducted a similar analysis on LD group size. We found that for five traits (A17 biomass, R108 biomass, annual mean temperature, streptomycin resistance, and spectinomycin resistance), the most strongly associated candidates were in larger LD groups than the most strongly associated variants from the random permutations (Fig. S5), suggesting that epistatic interactions may contribute to variation in these traits. Last, we compared the relative importance of SNPs and PAVs, by counting the number of SNPs and PAVs among the 10 LD groups most strongly associated with phenotypic variation. Although it is likely that not all ten of the candidates we analyzed for each trait truly underlie phenotypic variation, inclusion of false candidates is expected to make the analysis conservative. For three traits (biomass of A17 and R108 hosts and annual mean temperature), these LD groups were strongly enriched for SNPs (*P* ≤ 0.01) relative to the empirical null distribution, and for five other traits the LD groups were slightly to moderately enriched for SNPs (*P* < 0.20) (Fig. S5). These results suggest that SNPs contribute more to naturally occurring phenotypic variation than PAVs.

10.1128/mSphere.00386-18.6 FIG S5 Size and composition (brown SNPs, blue PAVs) of top 10 LD groups from the testing of the real phenotype data (column 1). (Column 2) The distribution of the mean proportion of SNPs in the top 10 LD groups from 100 GWA runs with randomly permuted phenotypes (LD groups containing both SNPs and PAVs were counted fractionally). The vertical line indicates the mean proportion of SNPs in the real data, and the percentage of random runs with a value greater than the real data is annotated to the right of the line. The genome-wide mean proportion of SNPs is 0.73. (Columns 3 and 4) The distribution of mean LD group size and mean MAF, respectively, for the permutations compared to the real data. Download FIG S5, PDF file, 1.3 MB. Copyright © 2018 Epstein et al. 2018 Epstein et al. This content is distributed under the terms of the Creative Commons Attribution 4.0 International license.

## DISCUSSION

Genome-wide association analyses provide a potentially powerful approach for identifying the functional role of naturally occurring allelic variation in bacterial populations (12, 14). As such, these analyses complement forward and reverse genetics as well as experimental evolution, all of which rely primarily on *de novo* mutation, for understanding gene function and genomic diversity. The association analyses we conducted on the model rhizobium Ensifer meliloti help to evaluate the potential success of these analyses in bacterial lineages and identified candidate genes that may underlie phenotypic variation in important ecological and agronomic traits.

### Diversity and linkage disequilibrium.

The potential success of association analyses depends on having a sample of strains that is genetically variable and on having individual variants that can be statistically distinguished, i.e., in which linkage disequilibrium (LD) is not extensive. Our sample exhibits extensive nucleotide and gene presence-absence variation (PAV), with more than 100,000 common (MAF > 0.05) SNPs and more than 10,000 genes with common PAVs, and a pangenome that contains nearly 67,000 genes. The number of SNPs used for association mapping here is comparable to other bacterial association studies—Earle et al. (18) identified 100,000 to 330,000 SNPs with MAF > 0.05 among 241 Escherichia coli, 176 Klebsiella pneumoniae, and 992 Staphylococcus aureus strains and Porter et al. (15) identified ∼200,000 SNPs in 47 *Mesorhizobium* strains. The level of gene presence-absence variation is especially high in our sample, though most of the variably present genes are found in only one strain—Earle et al. (18) found only ∼15,000 to 25,000 genes in the pangenomes of their samples, even though their S. aureus sample was much larger than our sample. The large pangenome of our strains may be the result of having a worldwide sample from a species that lives in both soil and hosts (37, 38).

Our sample also shows clear evidence of historical recombination, with mean genome-wide *r*^2^ of ∼0.06, and *r*^2^ ≤ 0.12 between variants on each of the megaplasmids (Table 1). The generally small size of LD groups (half contain three or fewer variants) means that association analyses have the potential to finely map variants responsible for phenotypic variation. Nevertheless, there are some large LD groups that limit the resolution of association mapping. Although there has been extensive work to characterize recombination rates in bacterial species (39), few studies have characterized genome-wide LD such as we have here, and thus, it remains unclear if the extent of LD we find in *Ensifer* is typical. As such, we cannot make any general statements about the expected complications of LD for conducting association analyses in bacteria. However, LD decays within 10 kb among closely related strains of S. aureus, and the median length of linkage blocks in two samples of Streptococcus pneumoniae was less than 200 bp (40), suggesting that fine-mapping of causative variants is possible in at least some other bacterial lineages. In other bacterial species or samples, such as Mycobacterium tuberculosis, which has very little recombination (41), samples of recombining bacteria with strong population structure (18, 42), and samples drawn from rapidly expanding epidemic populations (26), LD will likely be too extensive to permit fine mapping of causal variants.

### Genetic architecture.

The twenty traits we used for association analyses were all phenotypically variable (Fig. S2); however, we did not detect a genetic basis for all of this variation. For approximately half of the traits, the proportion of phenotypic variance explained by all variants (Fig. S4) or the most strongly associated variants (Fig. S3) was well within the empirical null distribution. Phenotypic variation in these traits is presumably due to microenvironmental variation during growth or assaying. That only half of the traits had strong support for harboring natural genetic variation underscores that prior to conducting association analyses it is valuable to estimate the proportion of phenotypic variation that is due to genotypic variation, i.e., heritability. All else being equal, the higher the heritability, the greater the power to link phenotype to genotype.

For the eleven traits for which there was evidence of genetic variation, the ability to identify specific SNPs or PAVs responsible for the phenotypic variation varied widely. For example, the mostly strongly associated variants explained a large amount of variation in A17 biomass and resistance to spectinomycin or streptomycin. In contrast, the most strongly associated variants for resistance to gentamicin and use of formic acid as a carbon source explained only slightly more variation than expected by chance (Fig. S3), even though the polygenic PVE analyses indicated a fairly strong genetic basis for these traits (Fig. S4). The disparity between variance explained by just the top variants and by all variants may indicate that variation in these traits is primarily the result of small effects by a large number of genetic variants, suggesting that highly polygenic traits are found in prokaryotes as well as eukaryotes (2). Alternatively, there may be large-effect alleles that are closely aligned with strain relatedness. In this case, the association analyses that we used, which remove the effect of relatedness before testing for the effects of individual variants, would be unable to detect the causative variants.

The characteristics of association candidates can provide some insight into genetic architecture and past selection. The candidates underlying variation in A17 biomass, the trait showing the strongest associations, were segregating alleles at a frequency much closer to 0.5 than is expected by chance (i.e., high minor allele frequency [MAF]) and were found as part of LD groups that are larger (i.e., include a greater number of variants) than expected by chance (Fig. S5). Candidates underlying variation in streptomycin and spectinomycin resistance, annual mean temperature, and R108 biomass also were members of LD groups that are larger than expected by chance (Fig. S5). High MAF is consistent with selection maintaining allelic polymorphism, possibly reflecting frequency-dependent or spatially variable selection acting on symbiosis genes (e.g., references 43 and 44). Candidates being part of large LD groups, in contrast, suggests epistatic interactions between multiple genes. Epistasis poses a challenge for association analyses because testing for pairwise interactions typically requires large sample sizes and greatly increases the computational burden while making it more difficult to filter out false associations and correct for multiple tests (1, 45). For prokaryotic lineages, in which epistasis can be particularly important (46) and long-distance LD blocks can be relatively easily identified, the number of variants in the LD groups provides a possible signature of epistasis. Such signatures may provide a means to characterize the extent to which epistasis contributes to phenotypic variation, even if it does not provide a means to identify the causative genes.

Interestingly, only one of the candidates we identified was found on the chromosome (Table 2), despite the fact that the chromosome comprises approximately half of the genome and harbors half of the genes. Chromosomal genes have been identified as being primarily involved in housekeeping functions (47), and the traits we characterized were primarily related to nonhousekeeping functions. However, it is possible that the lack of variation is biased by phenotyping being conducted in single-strain, noncompetitive environments; approximately half of the variants identified by a “select-and-resequence” experiment as underlying variation in nodulation ability in competitive conditions were found on the chromosome (48).

### Candidate genes.

Association analyses were developed to identify the genes and alleles responsible for phenotypic variation. The candidates we identified (Table 2) included genes previously identified through forward genetic approaches as well as genes without previously identified functions. In particular, several of the SNPs associated with plant biomass are in genes known to be important for symbiotic nitrogen fixation. These include *nifA*, which regulates the expression of nitrogenase genes (49); *fixN*, which is part of a cytochrome *c* oxidase that is necessary for respiration under the low-oxygen conditions in nodules (50); and *queC*, which is necessary for queuosine production (51), which is essential for effective N-fixing symbiosis (52). Other genes associated with plant biomass have not been previously shown to be directly involved in symbiosis but have functions that may be linked to symbiosis or nitrogen fixation. These included a diguanylate cyclase/phosphodiesterase, which regulates c-di-GMP levels, which in turn affect cell surface polysaccharides (53, 54); *gabD*, which is highly expressed in Rhizobium leguminosarum nodules (55) and may play a role in energy production (56); and a sulfotransferase. Sulfotransferases are necessary for the synthesis of effective nitrogenases and can modify nod factors, which affect interactions with legume hosts (e.g., reference 57).

The genes discussed above are potentially responsible for variation in the benefit rhizobia provide to their hosts when there are not any other strains present. A recent study using the same plant genotypes and many of the same *E. meliloti* strains used a select and resequence (“S and R”) approach to identify genes potentially responsible for variation in the ability of rhizobia to extract benefits from the host under competitive conditions (48). Interestingly, while both studies identified known and novel candidates for rhizobium-legume interactions, the list of genes only partially overlapped. Both studies identified *queC* and *nifA* as candidates, and the select and resequence study identified copies of diguanylate cyclase/phosphodiesterase and a cytochrome *c* oxidase subunit 1 that were different from the copies identified here, as well a gene in the same cytochrome *c* complex as the *fixN* identified here. In addition, the *fixL* and *fixJ* genes, which regulate *fixN* (49), were identified by the S and R study, though the top SNPs in these genes were not in the top 100 LD groups in this study. While both studies identified N-fixation-related candidates, only the S and R study identified candidates related to motility (e.g., flagellin B) or communication with the host (e.g., exopolysaccharide production). Thus, variants that make a rhizobium good at competing for limited nodulation opportunities are not necessarily the same variants that make a rhizobium good at fixing nitrogen for the host.

In contrast with the host benefit traits, our association analysis did not identify any genes with functions related to mechanisms previously implicated in resistance to the three aminoglycoside antibiotics we studied. These mechanisms include alteration of the ribosomal target through mutations in *gidB*; aminoglycoside modification by N-acetyltransferases, O-adenyltransferases, or O-phosphotransferases; and reduced uptake or increased export of the antibiotic (reviewed in reference 58). Instead, for spectinomycin, we found associations with the presence of a gene of unknown function; for streptomycin, a gene encoding a signal transduction protein; and for gentamicin, SNPs in genes encoding a ROK family regulatory protein, a nitrogenase iron protein (*nifH*), and an aldehyde dehydrogenase (Table 2). Association analyses might not find associations with previously identified genes because these genes are not segregating functionally important variants in natural populations. However, in *gidB* there were 13 common (MAF, >5%) SNPs segregating in our sample, although none of these changed amino acid residues that are known to affect streptomycin resistance in E. coli (60), and the associations with resistance were weak (all *P* > 0.001 compared to *P* < 0.00003 for top candidates, and all rankings >400). Similarly, we found six common PAVs annotated as aminoglycoside resistance genes, but these also were not strongly associated with aminoglycoside resistance (all *P* > 0.05), and all strains carried at least three genes annotated as aminoglycoside resistance genes. That we did not detect strong associations with previously identified resistance genes underscores the need to view genome annotations with caution and suggests that there may be unidentified, naturally occurring mechanisms of aminoglycoside resistance. It is also possible that some of the genes that do show strong associations are statistical false positives. For example, it is surprising that variation in *nifH*, a gene that encodes part of the nitrogenase enzyme, is found to be associated with antibiotic resistance rather than nodule number or plant biomass. The genes we identified should be viewed as candidates for further functional characterization. They may not contain the causative alleles but rather be in LD with causative alleles that did not meet our criteria for testing.

The five other phenotypes have not been well studied previously, and thus, there are not strong *a priori* candidates. Nevertheless, it is striking that for three of these phenotypes nitrogen fixation or metabolism genes (*fixI* for 2-aminoethanol utilization, *napA* for formic acid utilization, and *fixS* for annual precipitation) are associated with phenotypic variation. GWAS can also be used to find genetic variants associated with the climate of origin (i.e., candidates for local adaptation). For instance, Yoder et al. (59) identified (and validated) genetic variants associated with temperature and moisture at the site of origin for one of the plant host species of *Ensifer*. Using a similar approach on the bacterial partner, we identified two candidates potentially underlying adaptation to temperature and precipitation.

### Conclusions.

The association analyses we conducted have identified strong genotype-phenotype associations for several ecologically important *E. meliloti* phenotypes, including benefit provided to host plants, antibiotic resistance, climate adaptation, desiccation tolerance, and use of several carbon sources. These results clearly demonstrate the potential power of association analyses in bacteria and suggest that linkage disequilibrium will not prevent resolving associations down to relatively small genomic regions. Given our success at identifying known and novel promising candidate genes for plant biomass, future association analyses in rhizobia may be able to map variation in more mechanistic traits, such as nitrogenase activity, for a deeper understanding of the basis of variation in legume-rhizobium interactions. Nevertheless, the strongest associations for several traits did not exceed expectations from the empirical null distribution, and several traits harbored no signal of a genetic basis for phenotypic variation, underscoring that even in the presence of phenotypic variation, association analyses may not be appropriate or will have limited power.

## MATERIALS AND METHODS

We analyzed 153 *E. meliloti* strains originally collected from throughout the world (see files in Dryad Repository [https://doi.org/10.5061/dryad.tn6652t/1]) using *Medicago* sp. trap plants. From each strain DNA was extracted using a MoBio UltraClean microbial DNA isolation kit (12224), used to prepare dual-indexed Nextera XT libraries, and sequenced using 300-bp paired-end runs on an Illumina MiSeq. Strains were sequenced to a depth of 9.5 to 36.9 reads per base (mean = 19.5, 270,000 to 730,000 reads) after trimming and alignment. Reads were trimmed with Sickle (61) using a quality score of ≥20 and minimum length ≥127 bp. Paired-end reads were aligned to *E. meliloti* USDA1106, a strain that is similar to Rm1021 but has ∼10% more coding sequence (62), using bwa mem (v0.7.17) (63) and SNPs were called using parallel FreeBayes v1.0.2-16-gd466dde (64), both with default settings.

The raw variant call output from FreeBayes was filtered using utilities from vcftools (v0.1.15) (65), bcftools (v1.3.1), and vcflib (available from https://github.com/vcflib/vcflib). In particular, we removed SNPs with quality scores <20 and all indels, split sites with more than two alleles into multiple biallelic entries (bcftools norm), converted heterozygous calls (∼1% of variants, 0 to 2.2% for individual strains) into missing data, and removed sites with missing genotype calls in more than 20% of the strains. Because FreeBayes sometimes treats adjacent SNPs as a single multinucleotide polymorphism (MNP), we retained each MNP as a single variant. For simplicity we refer to both MNPs and SNPs as SNPs. Only the ∼110,000 SNPs with minor allele frequency (MAF) ≥ 5% were included in association analyses. We characterized the extent of nucleotide variation using two standard measures, Watterson's theta (θ_W_)—an estimate of the number of variants per bp (66)—and θ_π_—a measure of the number of pairwise variants per bp (67). These diversity statistics were calculated using the libsequence analysis package (v0.8.2) (68) on all sites with ≥2× coverage from ≥80% of the strains.

We identified presence-absence variants (PAVs) using *de novo* assemblies of each strain. The assemblies were constructed using SPAdes (v3.6.2) (69) using recommended settings, and genes were predicted using Glimmer (3.0.2) (70) and annotated using InterProScan (71). After omitting genes that were exceptionally long (≥5,000 bp), we clustered genes using CD-HIT (cd-hit-est; v4.6.8) (72, 73) with minimum identity set to 90% and the -aL and -AL parameters (amount of sequence that must be included in the match) set to 70%. Each of the 66,989 CD-HIT clusters was treated as a gene and considered present in a strain if that strain had one or members in the cluster. We conducted the LD grouping and association analyses using the 110,603 SNPs and the 13,352 PAVs with MAF ≥ 0.05.

### Phenotype measurements.

We conducted association analyses on 20 traits selected from 180 traits on which we had initially collected phenotypic data. The 180 traits included 95 quantitative Biolog plate traits (Biolog, Hayward, CA, USA); 78 “binary” antibiotic resistance, stress tolerance, enzymatic activity, and toxin tolerance traits; growth rate in liquid TY medium; two climatic variables describing where the strains were collected; and four symbiosis traits. A full description of phenotyping methods is provided in the supplemental methods (Text S1), and full trait data are in Dryad Repository (https://doi.org/10.5061/dryad.tn6652t/1).

10.1128/mSphere.00386-18.1 TEXT S1 Supplemental methods describing phenotype measurement methods in detail. Download Text S1, PDF file, 0.1 MB. Copyright © 2018 Epstein et al. 2018 Epstein et al. This content is distributed under the terms of the Creative Commons Attribution 4.0 International license.

We used a series of criteria to select the 20 traits for association analyses. First, we excluded Biolog and binary traits for which there was no or very little variation. This included 16 Biolog traits with variance < 0.01 (three environments with in which no strains grew and 13 sugar or sugar-alcohol utilization traits that were correlated with sucrose, glucose, and fructose utilization and for which all the strains grew vigorously), approximately 50 binary traits for which fewer than five strains were found at each phenotype value, and 50 traits for which <10% of the phenotypic variance was attributed to genetic differences among strains (PVE), as determined by a Bayesian sparse linear mixed model (BSLMM (35), implemented in GEMMA v0.94.1 (74). From the remaining 56 traits, we picked 10 that were not strongly correlated (Pearson’s r < 0.7) and had potential environmental relevance (e.g., antibiotic resistance) or a link to biochemical pathways involved in symbiosis, nitrogen metabolism, or ecology (use of l-fucose, 2-aminoethanol, *N*-acetylglucosamine, putrescine, and formic acid as carbon sources). We then added the four symbiosis traits (nodule number and plant biomass in two plant genotypes), two climate variables (Fig. S6), growth rate in liquid culture, and tolerance of high temperatures, desiccation, and salt. For additional information on the traits, including sample sizes, summary statistics, and a brief description, see Table S1.

10.1128/mSphere.00386-18.7 FIG S6 Positions of strains and loadings of the 19 core bioclim variables on the first four PCA axes (variance explained in parentheses). Precipitation-related variables are shown in blue, and temperature-related variables are shown in red. Annual mean temperature (AMT) and annual precipitation (AP) were chosen as the representative variables for further analysis. Variable abbreviations: AMT, annual mean temperature (temp.); MDR, mean diurnal range; IT, isothermality; TS, temp. seasonality; TWaM, max. temp. warmest month; TCM, min. temp. coldest month; TAR, temp. annual range; TWeQ, mean temp. wettest quarter; TDQ, mean temp. driest quarter; TWaQ, mean temp. warmest quarter; TCQ, mean temp. coldest quarter; AP, annual precipitation (precip.); PWeM, precip. wettest month; PDM, precip. driest month; PS, precip. seasonality; PWeQ, precip. wettest quarter; PDQ, precip. driest quarter; PWaQ, precip. warmest quarter; PCQ, precip. coldest quarter. Download FIG S6, PDF file, 0.3 MB. Copyright © 2018 Epstein et al. 2018 Epstein et al. This content is distributed under the terms of the Creative Commons Attribution 4.0 International license.

10.1128/mSphere.00386-18.9 TABLE S1 Descriptions of the 20 phenotypes selected for association analyses. The phenotypes are listed by category. For binary traits, instead of a mean and standard deviation, the number of resistant strains (strains that grew) and the number of susceptible strains (strains that did not grow) are reported (resistant/susceptible in the Mean column). Download Table S1, PDF file, 0.1 MB. Copyright © 2018 Epstein et al. 2018 Epstein et al. This content is distributed under the terms of the Creative Commons Attribution 4.0 International license.

### LD grouping.

To identify variants in strong LD, we (i) sorted all variants by MAF, (ii) used the variant with highest MAF and fewest ungenotyped individuals as a “seed” for an LD group, and (iii) grouped all variants in LD with the seed. Steps 2 and 3 were repeated, using the next ungrouped variant as a seed. To reduce computational time and eliminate unnecessary statistical tests, we performed association analyses (below) on only the seed variant from each group, but then annotated groups based on all variants in the group (source code used to create LD groups in Dryad Repository, https://doi.org/10.5061/dryad.tn6652t/1). We formed groups with two LD thresholds, *r*^2^ ≥ 0.95 and *r*^2^ ≥ 0.80.

### Association analyses.

We tested for an association between variants and phenotypic variation using a linear mixed model (LMM) implemented with the -lmm option in GEMMA (v0.94.1) (74). We included a standardized K-matrix (a measure of pairwise relatedness between strains calculated using the -gk 2 option), as recommended in the GEMMA manual, to lessen the effects of unequal relatedness among strains. *P* values were calculated with a likelihood ratio test (-lmm 4 option).

There are several statistical biases that should be considered when interpreting results from association analysis (75). One bias is that the apparent phenotypic effects from strongly associated variants will be inflated because variants with overestimated effect sizes are more likely to show strong associations (i.e., the “Winner's Curse”). Association analyses also have greater power to detect large effect and common loci relative to small effect and rare loci, respectively. To provide insight into the magnitude of the effect of these biases, and to determine a significance cutoff value, we conducted association analyses on randomly permuted data in which the phenotype values were randomly assigned to the genotype. These permutation analyses provide an empirical null distribution to determine whether variation explained in the actual data is greater than expected by chance (36, 76).

We estimated the proportion of phenotypic variance explained by genetic variants (PVE) using three methods. First, to estimate the PVE explained by top variants, we performed linear regression with forward model selection (implemented with base R [77] functions) with the phenotype as the response and with 1 to 25 most strongly associated variants from the trait-specific association tests as the predictors. We also regressed each variant on the residuals from the previously chosen variants to get the proportion of remaining variance explained (PRVE). To identify candidate genes, we iterated through the top variants as ordered by the model selection step, stopping when we reached a variant that explained less remaining variance than the 95th percentile of the empirical null distribution. To determine the total PVE by top variants (Table 3), we identified the model with the largest cumulative PVE after subtracting the median PVE of the null distribution.

Second, we calculated the variance explained by just the K-matrix (the LMM “null model” estimate from GEMMA BSLMM log files)—i.e., the amount of phenotypic variance that can be explained by among-strain relatedness. This estimate is analogous to heritability, with a value of one indicating that all phenotypic variation can be explained by the patterns of relatedness among strains. Third, we used a Bayesian sparse linear mixed model (BSLMM) implemented in GEMMA to estimate PVE. BSLMM fits a model with a few variants with larger effects in addition to the background effects captured by relatedness. The analysis was run with the default options, 2.5-million-step burn-in, and 6 million steps (continuous phenotypes), 25 million steps (most binary phenotypes), or 80 million steps (Cd tolerance) after the burn-in. A preliminary analysis indicated that 6 million steps was sufficient for continuous phenotypes, but binary phenotypes needed more steps to converge. The estimates were recorded every 500 steps, and we combined the results of 5 independent chains for each phenotype. The distributions of the hyperparameters indicated that nearly all chains converged.

### Data availability and accession number(s).

Sequence data were deposited in the NCBI Sequence Read Archive (SRA) under accession numbers SRR6055493 to SRR6055666 (https://www.ncbi.nlm.nih.gov/sra) under BioProject PRJNA401434. Computer code, phenotype data, and full results files have been deposited in Dryad (https://doi.org/10.5061/dryad.tn6652t/1).

10.1128/mSphere.00386-18.8 FIG S7 QQ plots for the *P* values from likelihood ratio tests in linear mixed model GWA. The five focal traits have red titles, and the eleven traits with PVE greater than expected by chance have bolded titles. The *P* value distributions for four of these 11 traits showed no evidence for inflation, five traits (2-aminoethanol utilization, annual mean temperature, R108 nodule number, and plant biomass on both plant genotypes) showed some mild inflation, and resistance to spectinomycin and streptomycin both showed substantial inflation; that is, the distribution of *P* values was strongly skewed toward lower (more significant) values. Download FIG S7, PDF file, 0.9 MB. Copyright © 2018 Epstein et al. 2018 Epstein et al. This content is distributed under the terms of the Creative Commons Attribution 4.0 International license.
