# Supplementary material for: Genome-Wide Association Analyses in the Model Rhizobium Ensifer meliloti
Source: mSphere. 2018 Oct 24;3(5):e00386-18. doi: 10.1128/mSphere.00386-18 (PMC6200981; doi:10.1128/mSphere.00386-18)
Supplement: FIG S1 [file sph005182667sf1.pdf]

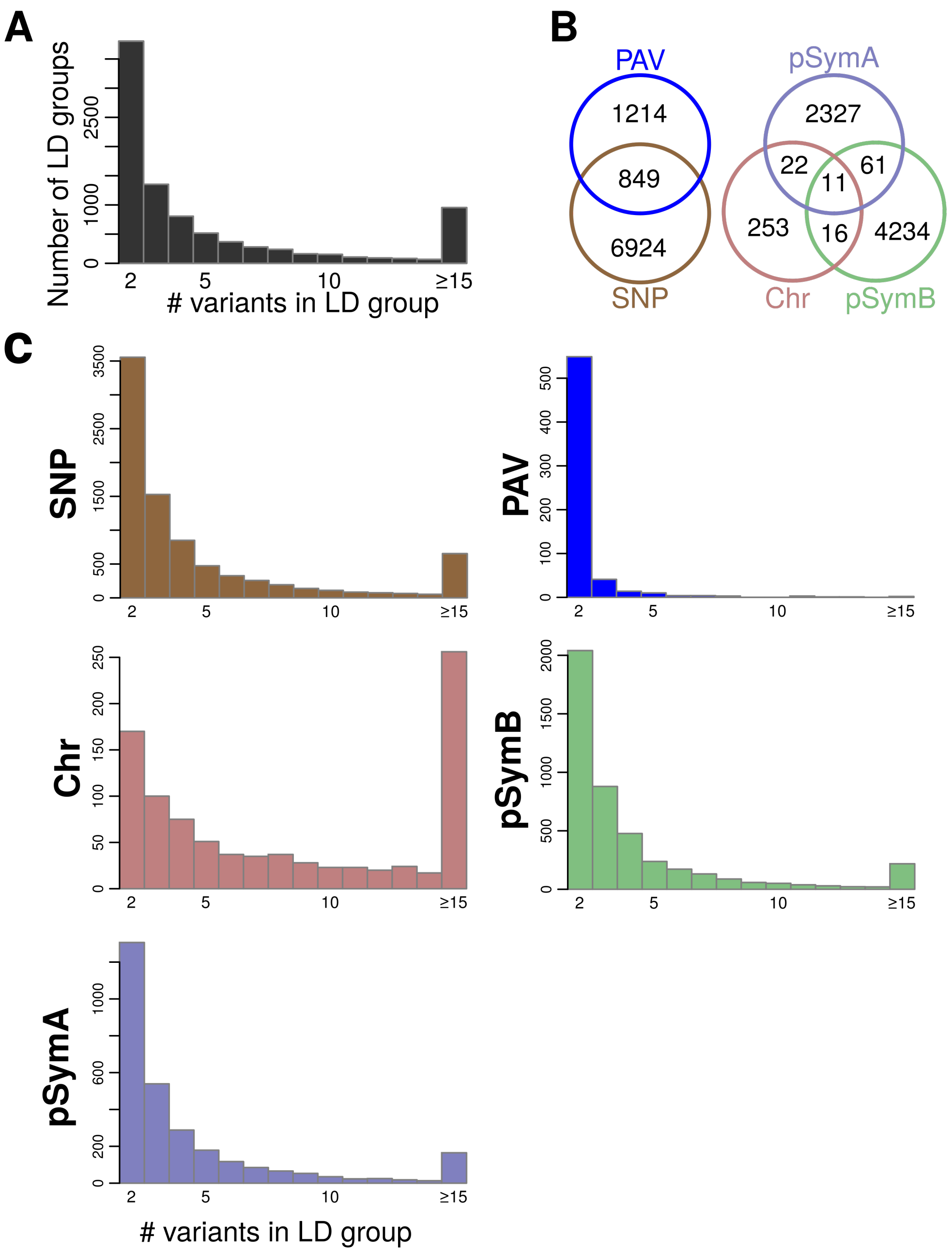
A
Number of LD groups
0
1000
2500
2
5
10
≥15
variants in LD group
B
PAV
1214
849
6924
SNP
pSymA
2327
22
61
11
253
16
4234
Chr
pSymB
C
SNP
PAV
Chr
pSymB
pSymA
variants in LD group

Fig. S1

The LD group size statistics are as follows:

Replicon	R^2	No. ungrouped variants	No. groups	Median number variants	Mean number variants	Max. # vars	N50	Median (median genomic span)	Median (max. genomic span)
All	0.80	18,522	8,987	3	11.7	6,979	37	N/A	N/A
All (SNPs)	0.95	22,057	8,364	3	6.3	857	12	N/A	N/A
All (PAVs)	0.95	10,764	632	2	2.4	31	2	N/A	N/A
Chrom.	0.95	789	900	7	16.3	454	37	51279.5	173405.5
pSymB	0.95	13,671	4,478	3	4.7	125	6	342.5	518
pSymA	0.95	7,597	2,912	3	5.7	857	9	635	1063

Replicon: Replicon and variant type

R^2: LD grouping threshold

No. ungrouped variants: Number of variants that were in their own group (not grouped with any other variants)

Median, Mean number variants: Of LD groups with more than one variant, the median and mean, respectively, number of variants in the groups

Max. # vars: The number of variants in the LD group with the most variants

Median(median genomic span): For each group that contained only SNPs, only SNPs on the same replicon, and had more than one variant, the median distance between pairs of variants was calculated. Then, the median across groups was reported in the table.

Median(max genomic span): Same as above, but the distance between the most distantly separated variants in each group instead of the median
