# Supplementary figures and images for: Genome-Wide Association Analyses in the Model Rhizobium Ensifer meliloti
Source: mSphere. 2018 Oct 24;3(5):e00386-18. doi: 10.1128/mSphere.00386-18 (PMC6200981; doi:10.1128/mSphere.00386-18)

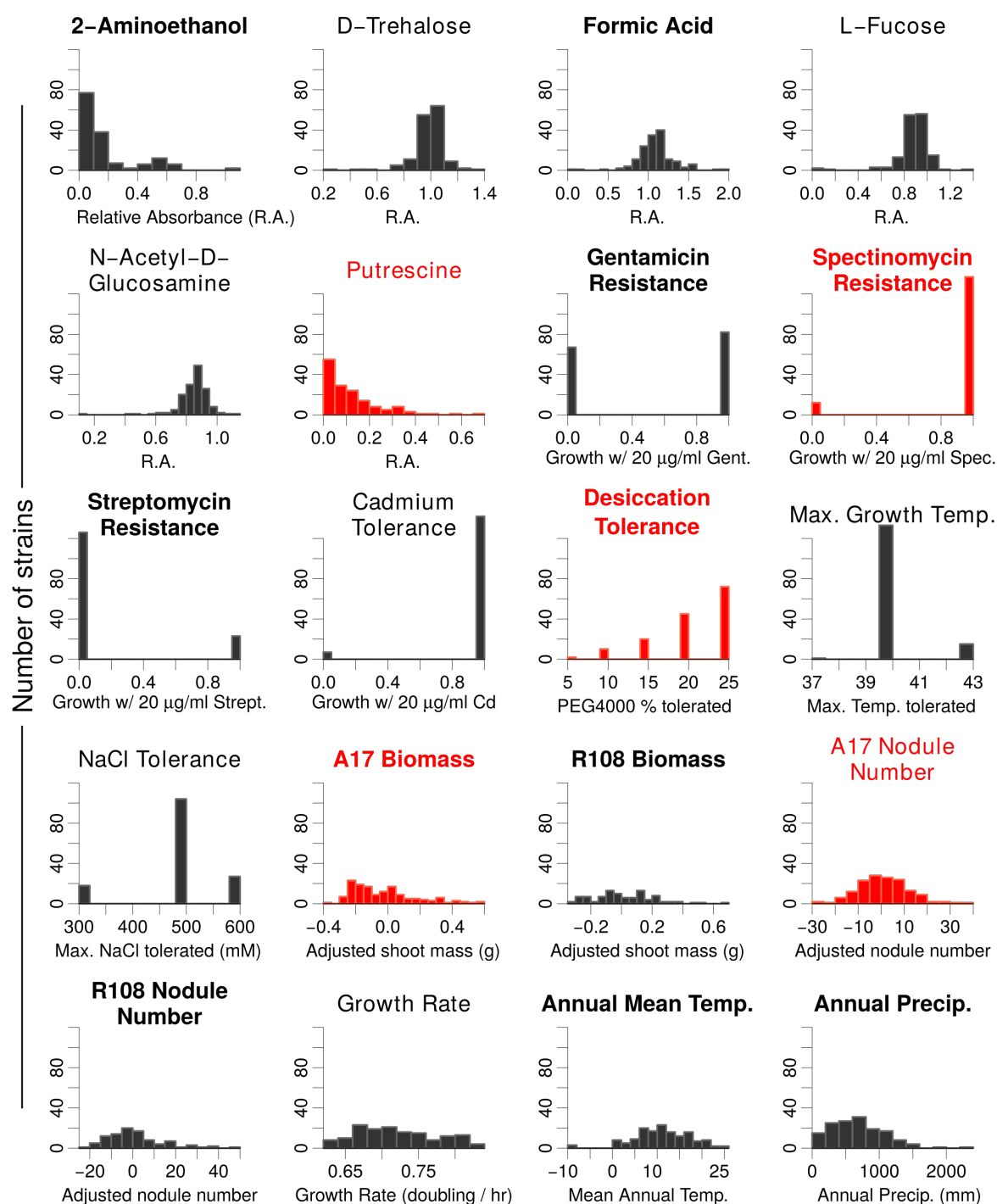

**Fig. S2**

Supplement: FIG S2 [file sph005182667sf2.pdf]

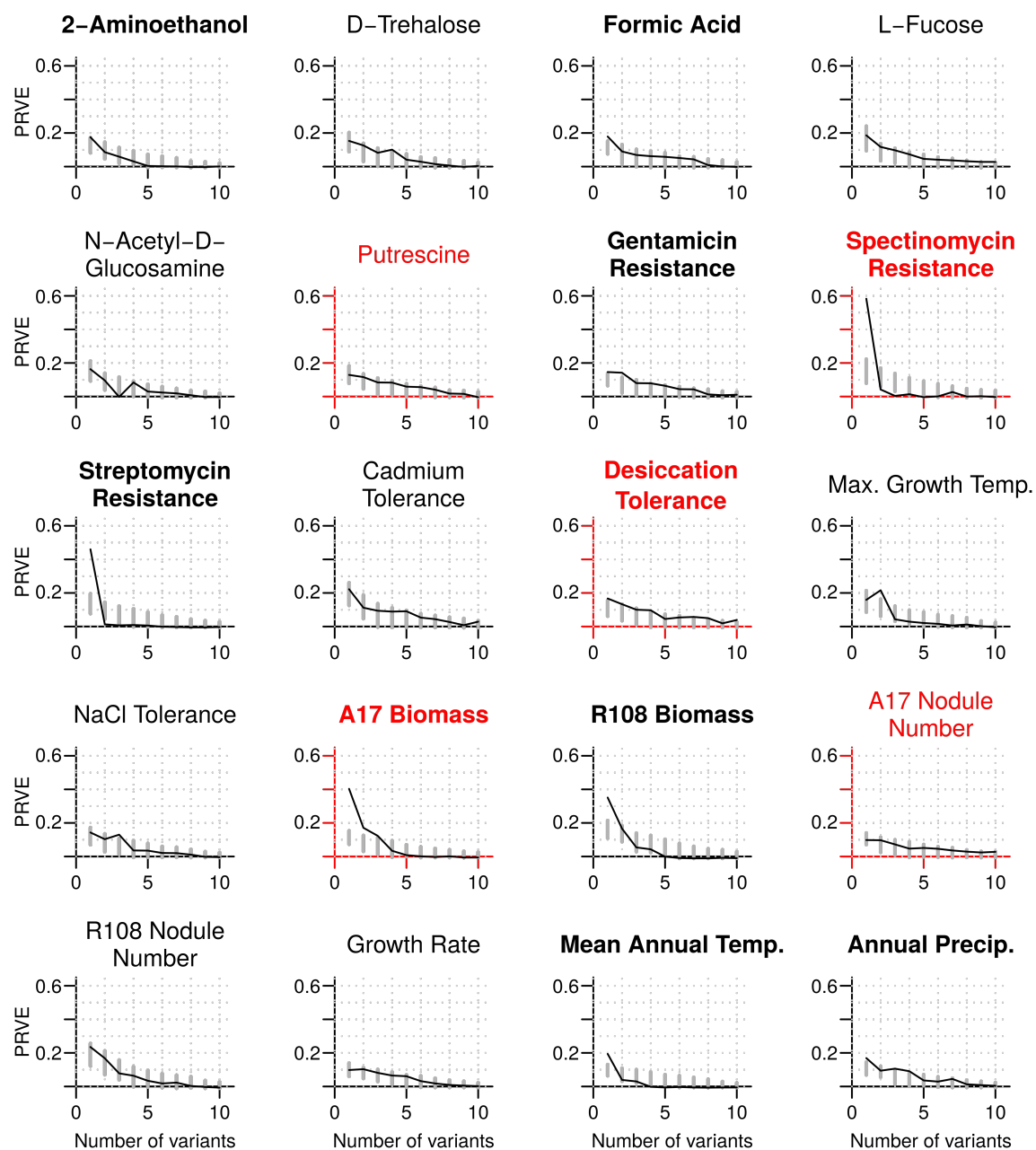

**Fig. S3**

Supplement: FIG S3 [file sph005182667sf3.pdf]

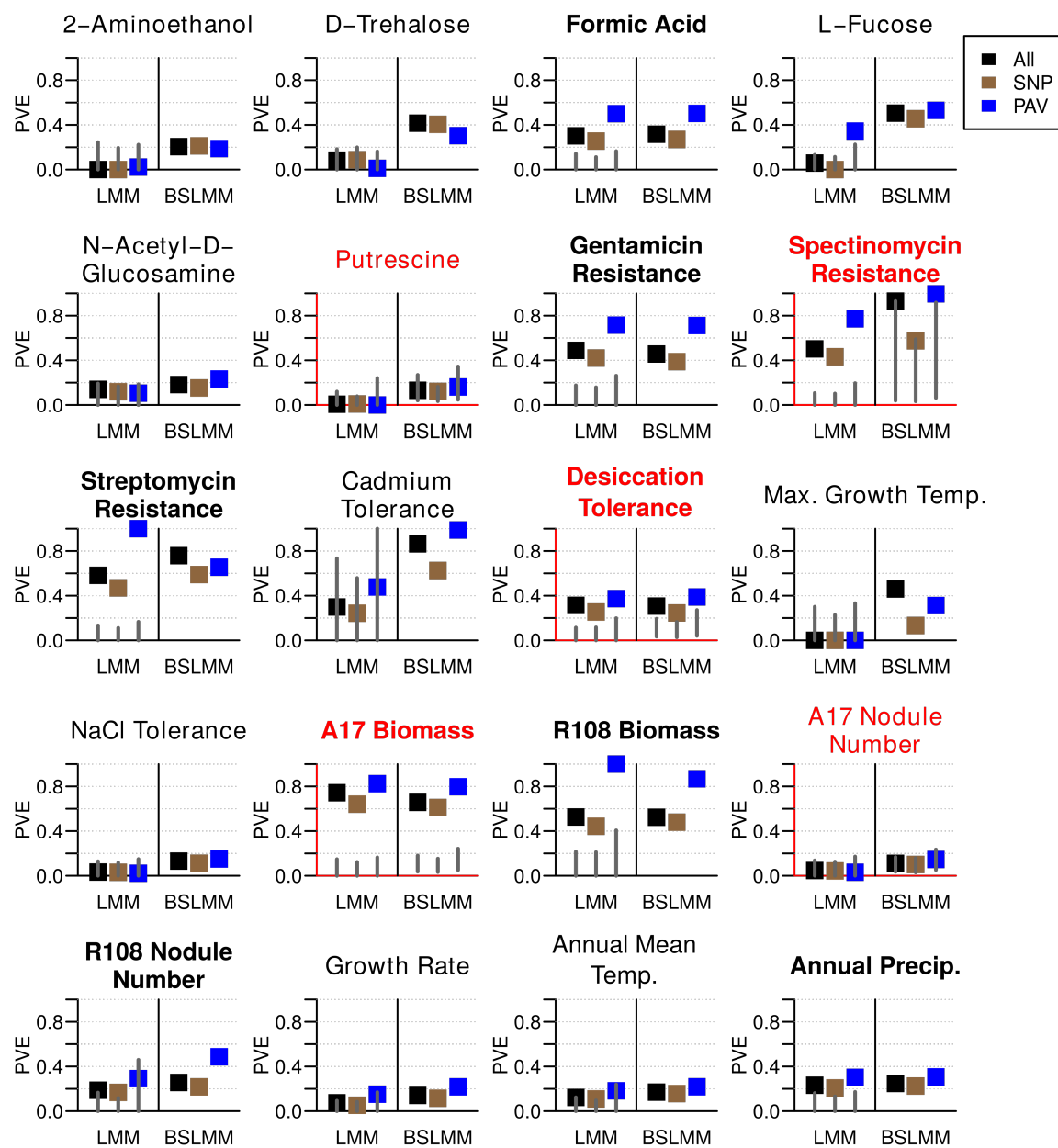

**Fig. S4**

Supplement: FIG S4 [file sph005182667sf4.pdf]

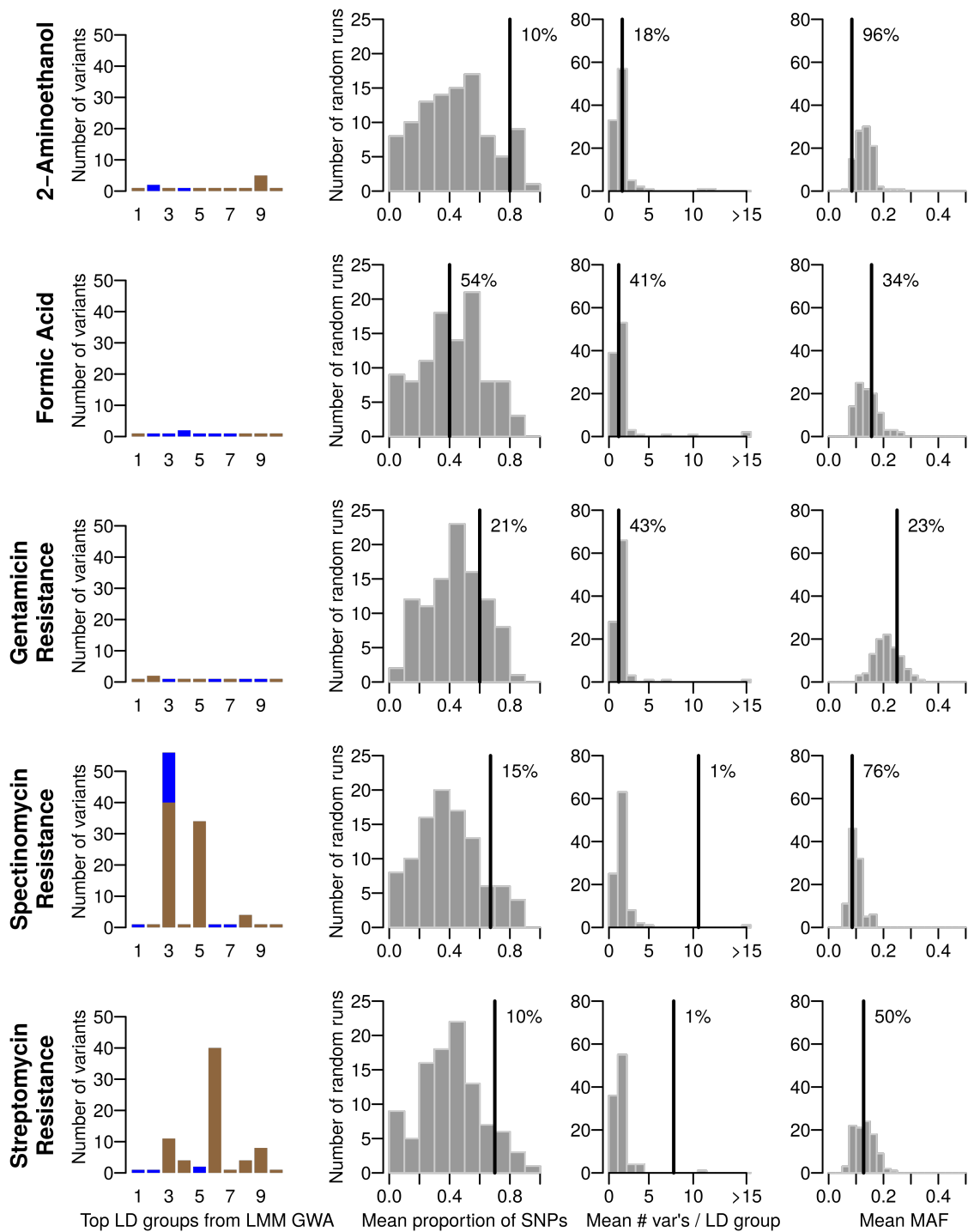

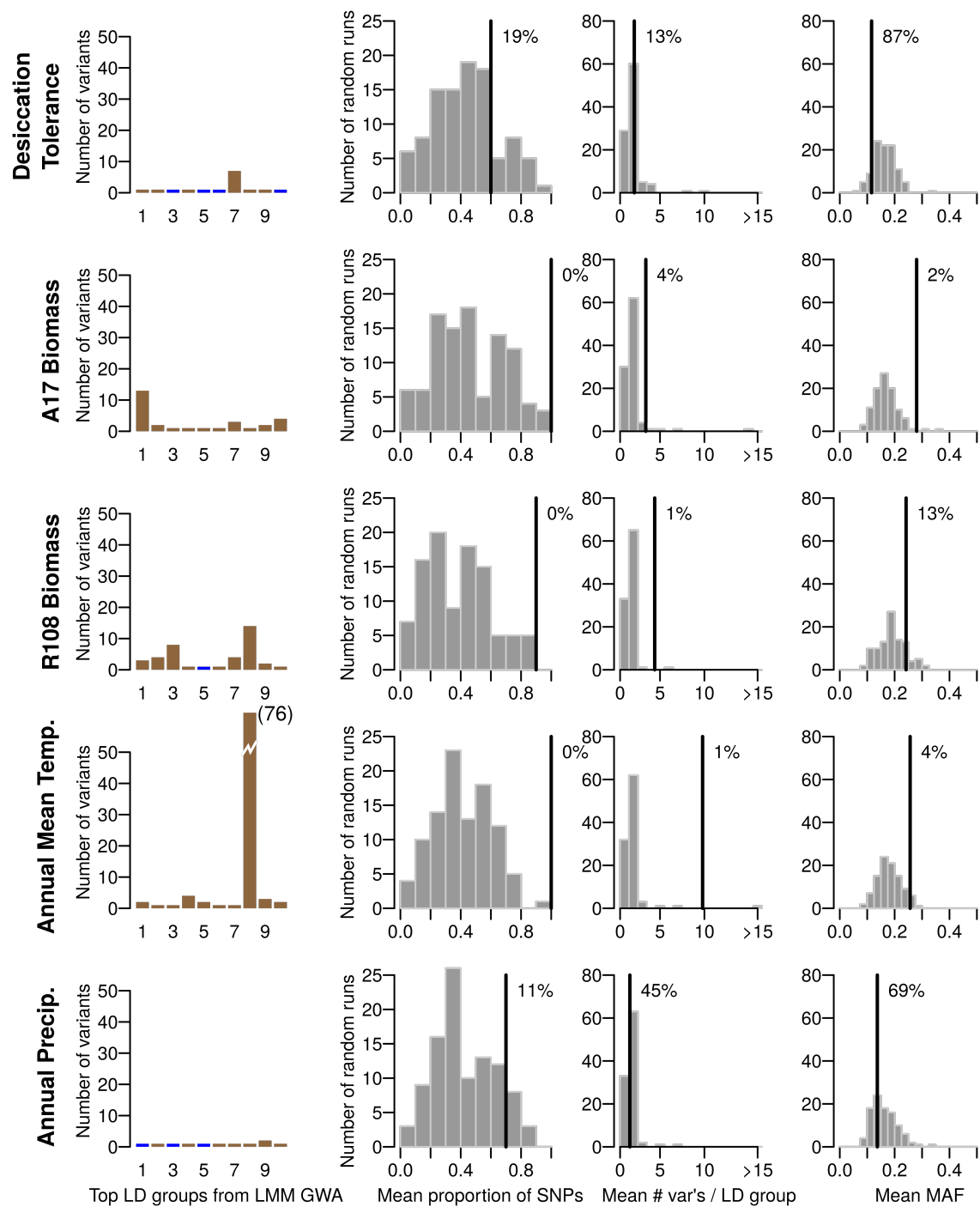

**Fig. S5**

Supplement: FIG S5 [file sph005182667sf5.pdf]

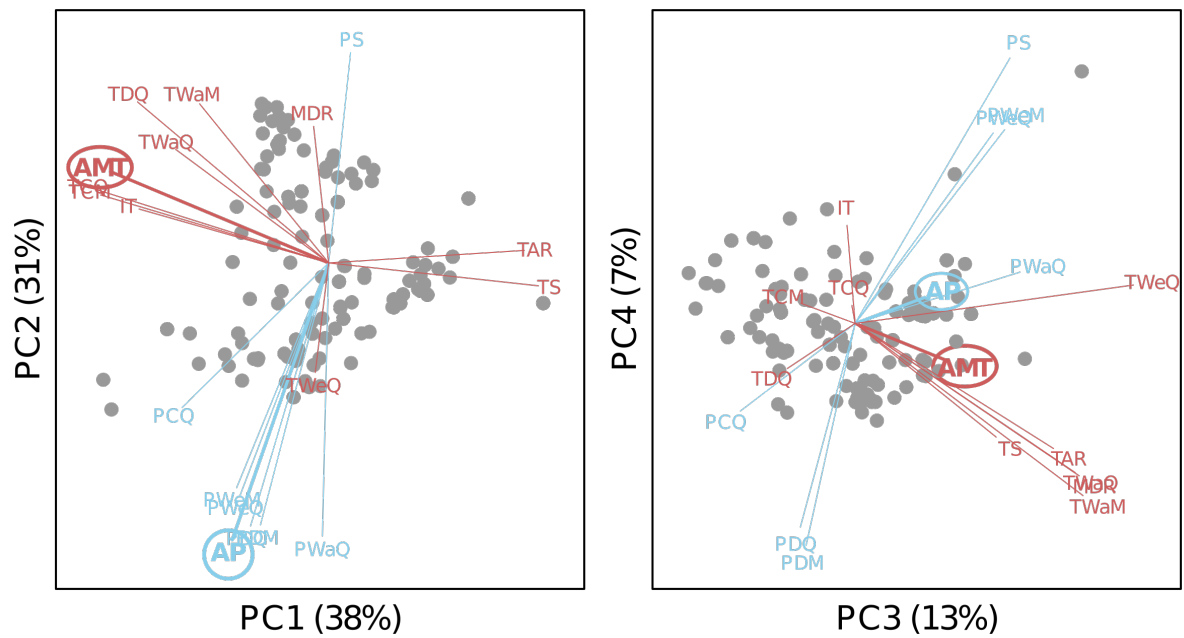

**Fig. S6**

Supplement: FIG S6 [file sph005182667sf6.pdf]
