# Supplementary material for: Genome-Wide Association Analyses in the Model Rhizobium Ensifer meliloti
Source: mSphere. 2018 Oct 24;3(5):e00386-18. doi: 10.1128/mSphere.00386-18 (PMC6200981; doi:10.1128/mSphere.00386-18)
Supplement: TEXT S1 [file sph005182667s1.pdf]

Text S1: Supplemental Phenotyping Methods

We conducted our analyses on 20 of the 180 traits for which we collected data. Here we describe, in detail, the methods used to measure these 20 phenotypes.

Biolog plates. Six of the 20 traits (2-aminoethanol, D-trehalose, formic acid, L-fucose, N-acetyl-D-glucosamine, and putrescine) were measured from Biolog GN2 plates (Biolog, Hayward, CA, USA); these plates contain 96 wells, each with a different carbon source and a dye that changes color in response to active bacterial metabolism. One plate per strain was inoculated according to the manufacturer's instructions, incubated at 30°C for 24 hours, and the absorbance at 540 nm, the wavelength at which the indicator dye absorbs, of each well in the plate was measured. To reduce among-plate and -batch variation in absorbance, we standardized the Biolog phenotypes by subtracting the absorbance of the blank and dividing by the mean absorbance of glucose, fructose, and sucrose. These three sugars could be metabolized by all strains, and in nearly all cases a well that contained one of these three sugars had greater absorbance than any other well on the plate.

Growth Rate. We measured growth rate in liquid TY media at 28° C in 96 well microtiter plates. Each well contained 200 µl of media, was inoculated with a single strain, and the OD_{600} was measured every 2 hours for 24 hours post-inoculation using the Synergy TM 4 Multi-Mode Microplate Reader (Bio Tek Instruments, Inc.). For each of three replicates, we made a plot of log(absorbance) vs. time, chose the part of the curve that was linear, and calculated generation time (G) as:

$$G = \frac{T_2 - T_1}{\log_2(OD_2) - \log_2(OD_1)}$$

where T_1 and T_2 are the beginning and end, respectively, of the log-linear part of the growth curve (in hours), and OD_1 and OD_2 are the absorbance values at T_1 and T_2, respectively (1). We used the mean reciprocal of generation time, across the three replicates, as growth rate.

“Binary” traits. Resistance to the antibiotics streptomycin, spectinomycin, and gentamicin, and tolerance of cadmium was assayed by inoculating TY plates amended with 20 µg $ml^{-1}$of one of the compounds, incubating at 30°C for 48 hours, and visually assessing whether there was growth (2). To assay strain ability to grow under water limiting conditions and salinity, we inoculated TY plates amended with

5%, 10%, 15%, 20%, and 25% PEG4000, or 200, 300, 500, 600, and 800 mM NaCl (3), and visually assessed growth after 48 hours at 30°C. To assess temperature tolerance, we incubated inoculated TY plates at 20, 28, 37, 40, and 43° C for 48 hours. All of these assays were carried out in triplicate and the response variable in each case was growth or no-growth. In some cases (12 out of > 2000 assays) the results were inconsistent among the three replicates, for these we used the most common result for the association analysis.

Symbiosis Traits. For a subset of the strains, we collected data on the number of nodules formed and the benefits they conferred to *M. truncatula* accession A17 (HM101, 150 strains) or R108 (HM340, 96 strains). To collect these data, we surface sterilized and scarified seeds before placing them on wet filter paper in the dark, overnight. Two seeds were planted into each autoclaved Leonard jar filled with 4:1 (v:v) Turface and Sunshine mix LP5, fertilized with N-free nutrient solution (4), inoculated with one ml of a culture (~ 10^8 cells) that was grown in liquid TY media at 30ºC for three days and washed with PBS (0.2 g/L K_2PO_4, 9 g/L NaCl, 0.73 g/L Na_2HPO_4•$7H_2O$). Leonard jars were randomly distributed between two growth chambers set at 22° C and 16 hours light / 8 hours dark. After five weeks, the plants were harvested, nodules were counted, and vegetative tissue was dried at room temperature and weighed. Each pot was treated as a replicate (1.8 mean replicates per strain). Due to practical constraints, planting was done in three batches, with each replicate coming from a different planting batch. To minimize variation among chambers or planting batch, we regressed (linear regression implemented in R v3.3.1) the phenotype values on batch, growth chamber, and the number of plants in each pot. From the residuals, we then took the mean of each strain/plant genotype combination, and used those values for association analyses. For example, the dry shoot mass in every Leonard jar was used as the response in a linear regression with batch, growth chamber, and the number of plants in the jar as predictors; after running the regression, the residuals (one value for each jar) were extracted, and the mean value for each host/strain combination was calculated. The raw values and the adjusted values were tightly correlated: A17 nodule number $r_{df=148} = 0.90$, R108 nodule number $r_{df=94} = 0.85$, A17 plant biomass $r_{df=144} = 0.90$, R108 plant biomass $r_{df=92} = 0.92$.

Climate of origin. We characterized the climate of the geographic locations the strains were originally sampled from by using climate data from the Bioclim 30 second resolution database (5) and performed a PCA using the prcomp function in R (v3.3.1; (6)) on variance-scaled and mean-centered data. We used the most precise description we could find from the USDA of the source of the strain to assign geographic coordinates; when the description included a large area (e.g. a country), we assigned the strain to the center of the area. All but 20 strains could be localized

to an area smaller than a country. The first two axes explained approximately 65% of the variance (PC1 ~34%, PC2 ~ 30%). Precipitation variables had high loadings on the second axis, while temperature variables had high loadings on either the first or second axis (Fig. S6). We chose annual mean temperature and annual precipitation as representative variables because they were among the top three loadings for the first or second axis, respectively.

References

1. Deshwal VK, Chaubey A. 2014. Isolation and characterization of *Rhizobium leguminosarum* from root nodule of *Pisum sativum* L. J Academ Industrial Res 2:464–467.

2. Josey DP, Beynon JL, Johnston AWB, Beringer JE. 1979. Strain identification in *Rhizobium* using intrinsic antibiotic resistance. J Appl Bacteriol 46:343–350.

3. Sadowsky MJ, Keyser HH, Bohlool BB. 1983. Biochemical characterization of fast-and slow-growing rhizobia that nodulate soybeans. Int J Syst Evol Microbiol 33:716–722.

4. Bucciarelli B, Hanan J, Palmquist D, Vance CP. 2006. A standardized method for analysis of *Medicago truncatula* phenotypic development. Plant Physiol 142:207.

5. Hijmans R, Cameron S, Parra J, Jones P, Jarvis A, Richardson K. 2005. WorldClim, version 1.3. University of California, Berkeley.

6. R Core Team. 2016. R: A language and environment for statistical computing. R Foundation for Statistical Computing, Vienna, Austria.
