# Supplementary material for: Genome-Wide Association Analyses in the Model Rhizobium Ensifer meliloti
Source: mSphere. 2018 Oct 24;3(5):e00386-18. doi: 10.1128/mSphere.00386-18 (PMC6200981; doi:10.1128/mSphere.00386-18)
Supplement: TABLE S1 [file sph005182667st1.pdf]

Table S1

Phenotype	N	Mean	Std. Deviation	Description
**Antibiotics**				
Gentamicin resistance	149	82 / 67	NA	Resistance to 20 µg / ml gentamicin
Spectinomycin resistance	149	137 / 12	NA	Resistance to 20 µg / ml spectinomycin
Streptomycin resistance	149	23 / 126	NA	Resistance to 20 µg / ml streptomycin
**Biochemical**				
2-Aminoethanol	150	0.17	0.21	Use of 2-aminoethanol as C source; 24 hr absorbance at 540 nm on Biolog GN2 plate after subtracting blank well and dividing by mean of fructose, glucose, and sucrose; ethanolamine is part of rhizobial lipopolysaccharides, the structure of which affect nodulation (1).
D-Trehalose	150	0.99	0.13	Use of D-trehalose as a C source; trehalose accumulation is important for osmotolerance (2); mutations in trehalose catabolism genes impact competition for nodule formation (3).
Formic Acid	150	1.09	0.24	Use of formic acid as a C source; formate can stimulate rhizobial N-fixation in culture (4).
L-Fucose	150	0.88	0.16	Use of L-fucose as a C source; fucosylation of nod-factors is involved in host specificity (5).
N-Acetyl-D-Glucosamine	150	0.85	0.11	Use of N-acetyl-D-glucosamine as a C source; N-acetylglucosamine forms the backbone of nod factors, which are essential for invasion of plant roots (6).
Putrescine	150	0.11	0.12	Use of putrescine as a C source; differential expression of host genes related to polyamine metabolism (7).
**Nodulation**				
A17 nodule number	150	36.2	11.9	Mean number of nodules formed on host genotype A17 five weeks after inoculation. The mean and std. dev. reported here are of the raw data rather than the adjusted data used for association analyses.
R108 nodule number	96	23.4	16.2	Mean number of nodules formed on host genotype R108.
A17 biomass	146	0.26	0.21	Mean dry mass (g) of plant genotype A17.
R108 biomass	94	0.27	0.22	Mean dry mass (g) of plant genotype R108.
**Environmental and Organismal**				
Growth rate	149	0.72	0.05	Mean number of doublings per hour, measured by OD_{600}, during the exponential growth phase in liquid tryptone-yeast (TY) media.
Desiccation tolerance	149	20.9	5.0	Maximum concentration of PEG4000 (%) that allowed growth on TY plates; values tested were 5%, 10%, 15%, 20%, and 25%.

Salt tolerance	149	494.0	81.6	Maximum concentration of NaCl (mM) that allowed growth on TY plates; values tested were 200, 300, 500, 600, and 800 mM.
Max. Growth Temperature	149	40.3	0.9	Maximum temperature (°C) that allowed growth on TY plates; values tested were 20, 28, 37, 40, and 43 °C.
Annual Mean Temperature	148	111.4	62.3	Annual mean temperature (°C x 10), according to the BioClim database, at the location from which the strain was collected
Annual Precipitation	148	678.8	393.7	Annual mean precipitation (mm) at the location from which the strain was collected
Cadmium tolerance	149	142 / 7	NA	Ability to grow on TY plates amended with 20 µg / ml Cd.

References

1. Kosenko L, Zatovskaya T. 2004. Investigation of lipopolysaccharides from *Sinorhizobium meliloti* SKHM1-188 and two of its mutants with decreased nodulation competitiveness. Microbiology 73:292–299.

2. Domínguez-Ferreras A, Soto MJ, Pérez-Arnedo R, Olivares J, Sanjuán J. 2009. Importance of trehalose biosynthesis for *Sinorhizobium meliloti* osmotolerance and nodulation of alfalfa roots. J Bacteriol 191:7490–7499.

3. Jensen JB, Ampomah OY, Darrah R, Peters NK, Bhuvaneswari T. 2005. Role of trehalose transport and utilization in *Sinorhizobium meliloti*-alfalfa interactions. Mol Plant Microbe In 18:694–702.

4. Manian SS, Gumbleton R, O'Gara F. 1982. The role of formate metabolism in nitrogen fixation in *Rhizobium Spp*. Arch Microbiol 133:312–317.

5. Stacey G, Luka S, Sanjuan J, Banfalvi Z, Nieuwkoop AJ, Chun JY, Forsberg LS, Carlson R. 1994. nodZ, a unique host-specific nodulation gene, is involved in the fucosylation of the lipooligosaccharide nodulation signal of *Bradyrhizobium japonicum*. J Bacteriol 176:620.

6. Jones KM, Kobayashi H, Davies BW, Taga ME, Walker GC. 2007. How rhizobial symbionts invade plants: the *Sinorhizobium–Medicago* model. Nat Rev Microbiol 5:619.

7. Burghardt LT, Guhlin J, Chun CL, Liu J, Sadowsky MJ, Stupar RM, Young ND, Tiffin P. 2017. Transcriptomic basis of genome by genome variation in a legume-rhizobia mutualism. Molec Ecol 26:6122–6135.
