# Supplementary material for: Genome-Wide Association Analyses in the Model Rhizobium Ensifer meliloti
Source: mSphere. 2018 Oct 24;3(5):e00386-18. doi: 10.1128/mSphere.00386-18 (PMC6200981; doi:10.1128/mSphere.00386-18)
Supplement: FIG S7 [file sph005182667sf7.pdf]

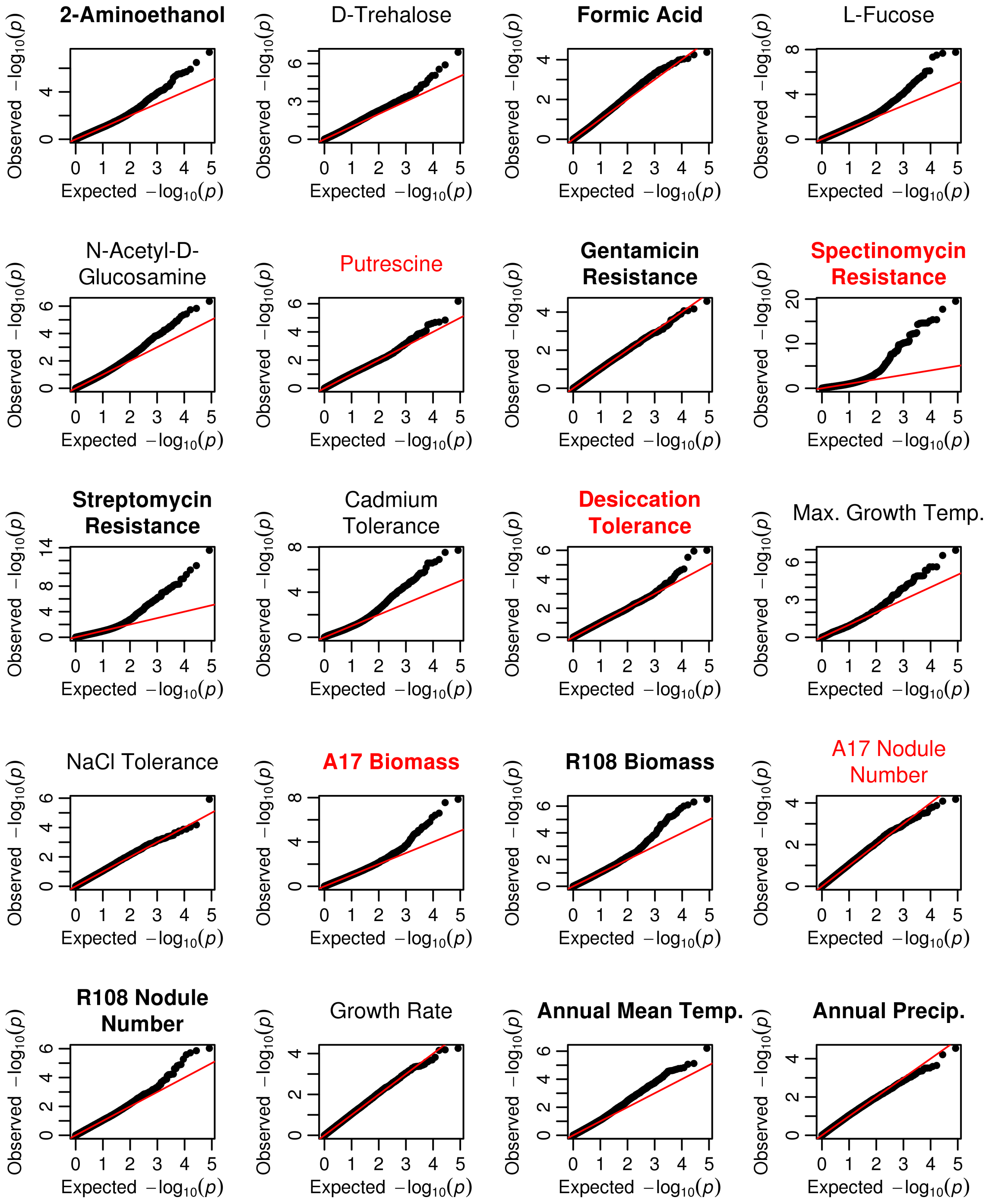

Fig. S7: QQ plots for the *p*-values from likelihood ratio tests in linear mixed model GWA. The five focal traits have red titles, and the eleven traits with PVE greater than expected by chance have bolded titles. The *p*-value distributions for four of these 11 traits showed no evidence for inflation, five traits (2-aminoethanol utilization, annual mean temperature, R108 nodule number, and plant biomass on both plant genotypes) showed some mild inflation, and resistance to spectinomycin and streptomycin both showed substantial inflation; that is, the distribution of *p*-values was strongly skewed towards lower (more significant) values.
